# LncRNA *EGOT* decreases breast cancer cell viability and migration via inactivation of the Hedgehog pathway

**DOI:** 10.1002/2211-5463.12833

**Published:** 2020-04-08

**Authors:** Shuang Qiu, Guobing Chen, Juan Peng, Jia Liu, Jumin Chen, Jianjun Wang, Li Li, Kunxian Yang

**Affiliations:** ^1^ Department of Breast and Thyroid Surgery The First People’s Hospital of Yunnan Province The Affiliated Hospital of Kunming University of Science and Technology Kunming China

**Keywords:** breast cancer, cell migration, cell proliferation, Hedgehog pathway, lncRNA *EGOT*

## Abstract

The long noncoding RNA (lncRNA) Eosinophil Granule Ontogeny Transcript (*EGOT*) has been reported to inhibit the proliferation and migration of glioma cells, and promote the development and progression of gastric cancer through the Hedgehog (Hh) signaling pathway. This study was conducted to assess the role of *EGOT* in the progression of breast cancer. We observed that *EGOT* is significantly down‐regulated in breast cancer tissues and cell lines, and *EGOT* expression is negatively correlated with the Ki67 expression. Overexpression of *EGOT* in BT549 cells decreased cell viability and migration. In addition, overexpression of *EGOT* resulted in decreases in expression of key genes in the Hh pathway, including Gli1, smoothened protein, protein patched homolog 1 and Hedgehog‐interacting protein (HHIP). Breast cancer tissues exhibited an increase in Gli1 expressions. Altered expression of Gli1, smoothened protein, protein patched homolog 1 and HHIP caused by *EGOT* overexpression were fully restored in cells transfected with plasmid complementory DNA (pcDNA) *EGOT* and treated with purmorphamine, an agonist of the Hh pathway. Cell viability and migration were also restored by purmorphamine. We conclude that lncRNA *EGOT* may inhibit breast cancer cell viability and migration via inactivation of the Hh pathway.

## Graphical abstract

Long noncoding RNA Eosinophil Granule Ontogeny Transcript (*EGOT*) is greatly down‐regulated in breast cancer tissues and cell lines. Furthermore, the expression of key genes in the Hedgehog (Hh) pathway is reduced at both mRNA and protein levels when *EGOT* is overexpressed. Purmorphamine restored the expression of Hh pathway genes and reversed the effects on cell viability and migration caused by overexpression of *EGOT*. *EGOT* thus may inhibit breast cancer cell viability and migration via inhibition of the Hh pathway.

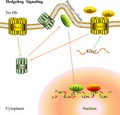

**Abbreviations**
*EGOT*: Eosinophil Granule Ontogeny Transcript
ER: estrogen receptor
HER‐2: human epidermal growth factor receptor 2
Hh: Hedgehog
lncRNA: long noncoding RNA
MTT: 3‐(4,5)‐dimethylthiahiazo(‐z‐y1)‐3,5‐di‐phenytetrazoliumromide
PVDF: poly(vinylidene difluoride)
qRT‐PCR: real‐time quantitative PCR
SD: standard deviation
TNBC: triple‐negative breast cancer

Breast cancer is a malignant tumor caused by a malignant tumor invasion and destruction of normal breast tissue. The global death toll of breast cancer accounts for 14% of all cancer deaths and is the second most common cancer among women. According to cancer statistics, there were more than 2.3 million new cases of invasive breast cancer worldwide in 2015, with 402 000 women having died of breast cancer [1]. In recent years, surgery, chemotherapy and radiotherapy have become the main methods of breast cancer treatment with the continuous development of various treatment methods [2]. However, 4–10% of patients with breast cancer in China are found with distant metastasis every year, with clinical treatments bringing poor results [3]. Early screening and treatment are both essential in preventing and helping to alleviate breast cancer.

Long noncoding RNA (lncRNA) is a type of noncoding RNA of more than 200 nucleotides, which is also a product of the RNA polymerase II transcription and lacks an ORF. The initial study concluded that lncRNA has biological functions. In recent years, studies have found that lncRNA can be involved in the development of tumors as a carcinogenic or tumor suppressor, with its abnormal expression being closely related to tumor cell proliferation, metastasis and apoptosis [4]. Aberrant expressions in lncRNA also play an important role in the development of breast cancer. Current studies have shown that HOTAIR [5], GAS5 [6], PVTl [7], MALATl [8] and other lncRNAs have strong effects on breast cancer cell proliferation, apoptosis and migration. The lncRNA Eosinophil Granule Ontogeny Transcript (*EGOT*), a human gene, is located in 3p26.1 and is highly conserved at the nucleic acid level. Wagner *et al.* [9] have found that *EGOT* is involved in the development of eosinophils and is expressed in mature eosinophils. They also have demonstrated that *EGOT* is not related to ribosomes and is likely to function as a noncoding RNA through sucrose density gradients. Wagner *et al. *[9] also additionally reported that *EGOT* is highly expressed in bone marrow and plays an important role in bone marrow hematopoietic stem cells. For some tumors, such as renal cell carcinoma, *EGOT* is a tumor suppressor and is likely a potential prognostic biomarker for kidney cancer [10]. Moreover, Wu *et al. *[11] have found that *EGOT* inhibits the proliferation and migration of glioma cells and promotes apoptosis in human gliomas.

The Hedgehog gene (Hh) was first discovered in *Drosophila* in 1980 [12] with the family of proteins having crucial functions in embryonic development and cell proliferation [13]. The Hh signaling pathway has been well known as an important signaling pathway and therapeutic target in various kinds of cancers [14]. The protein patched homolog 1 (PTCH) receptor prevents high expressions and activity of the smoothened protein (SMO) without the Hh ligand, whereas the repression of SMO is relieved when Hh is bound, which thus leads to the activation of glioma‐associated oncogene (GLI) transcription factors. These include activators Gli1 and Gli2, along with repressor Gli3. Activated GLI then controls transcriptions of Hh‐targeted genes [15, 16]. GLI can also be activated in a nonclassical way without the Hh ligand and SMO, via receptors in tumor‐associated cytokines. This largely occurs with transforming growth factor β and stromal‐derived factor 1 [17].

Peng *et al. *[18] have demonstrated that *EGOT* promotes the development and progression of gastric cancer through the Hh signaling pathway and can be used as a biomarker for the diagnosis and prognosis of gastric cancer. For breast cancer in particular, Xu *et al. *[19] point out that a down‐regulation of *EGOT* is associated with malignancy and a poor prognosis of breast cancer through clinical breast cancerous tissues and adjacent noncancerous tissues. They suggest, moreover, that antisense intronic lncRNA *EGOT* enhancing autophagy has sensitized paclitaxel cytotoxicity via an up‐regulation of ITPR1 expression by RNA–RNA and protein–RNA interactions in breast cancer [20]. Whether *EGOT* aberrant expressions have an effect on biological activities through Hh signals in breast cancer cells is still unclear.

The aim of this study, therefore, has been to confirm the expression pattern of *EGOT* in breast cancer cells and adjacent tissues to explore the effects of abnormal expressions in lncRNA *EGOT*, along with the viability and migration of breast cancer cell lines and the Hh signaling pathway.

## Materials and methods

### Tissue collection

From October 2016 to July 2018, a total of 50 paired breast tumor and adjacent normal tissues were collected after surgery at The First People’s Hospital of Yunnan Province, China. Tissues were washed in PBS and frozen in liquid nitrogen before being stored at −80 °C. Each patient provided a signed informed consent form. This study was approved by the Ethics Committee of The First People’s Hospital of Yunnan Province. The study conformed to the guidelines set by the Declaration of Helsinki.

### Cell lines and culture

Breast cancer cell lines, including BT549, MDA‐MB‐231, MCF7, SKBr3 and HEK293, were purchased from ATCC (Balimore, MD, USA). BT549 cells were cultured with RPMI 1640 (Gibco, Thermo Fisher Scientific, Inc, Grand Island, NY, USA) containing 10% FBS. MDA‐MB‐231 and SKBr3 cell lines were cultured in Dulbecco’s modified Eagle’s medium (Gibco, Thermo Fisher Scientific, Inc.) with 10% FBS. MCF‐7 was cultured in minimum Eagle's medium (MEM) including 10% FBS, and HEK293 was also cultured in MEM with 10% FBS. All of the cells were cultured in the constant temperature and humidity chamber at 37 °C with 5% carbon dioxide.

### Plasmids, cell transfection and purmorphamine treatment

pcDNA–EGOT plasmids were purchased from GenePharma (Shanghai, China). Plasmids were transfected into BT549 cells using Lipofectamine 2000 (Invitrogen, Carlsbad, CA, USA) according to the manufacturer’s protocol. Purmorphamine (120933; Abcam, Cambridge, UK), the agonist of the Hh pathway, was dissolved in DMSO for this project; the substance was then formulated into a 1 μm solution. After transfection with pcDNA–EGOT or pcDNA (empty vector), cells were treated with 1 μm purmorphamine of DMSO, and treatment with DMSO served as a control.

### MTT assay

After cells were counted, cell density was adjusted to 1 × 10^5^ cells·mL^−1^ with a serum‐free medium. Cells were added to a 96‐well plate and then cultured with 5% CO_2_ at 37 °C for 24, 48, 72 and 96 h, respectively. 3‐(4,5)‐Dimethylthiahiazo(‐z‐y1)‐3,5‐di‐phenytetrazoliumromide (MTT) was configured as a 5 mg·mL^−1^ solution in PBS, and 10 μL was added for incubation another 4 h; the absorbance (*OD*) value was further read at 490 nm by a microplate reader [21].

### Migration Transwell

Cells were collected and prepared into a cell suspension of 1 × 10^5^ cells·mL^−1^. A 600‐μL medium containing 10% FBS was added to the lower chamber, whereas a 200‐μL cell suspension was added to the upper chamber and incubated for 24 h; the liquid from the upper and lower chambers was then discarded. The cells of the lower chamber were fixed by 4% paraformaldehyde for 30 min. After this, the paraformaldehyde was removed, and the cells that did not pass through the membrane were wiped clean with a cotton swab. The lower chamber was stained with 0.1% crystal violet for 10 min, whereas the chamber was washed three times with PBS. Migrated cells were then observed and counted under a microscope [22].

### Wound healing assay

Horizontal lines across the well were drawn with a marker pen and ruler on the back of a six‐well plate, while every well passed at least five times. Cell suspensions at the concentration of 1 × 10^6^ were prepared, and 1 × 10^5^ cells were added to each well. Vertical lines to the back lines were drawn with the smaller pipettor and a ruler. Cells were then washed three times with PBS solution, while floating cells were removed completely and serum‐free medium was added. These cells were cultured at 37 °C with 5% CO_2_ for 24 h; after this, pictures were taken for examination purposes [23].

### Real‐time quantitative PCR

RNA extraction in the tissues and cells was performed by TRIzol reagent (Invitrogen) based on the manufacturer’s instructions, with the concentration of RNA being measured by the nanodrop. Genomic DNA was removed, and reverse transcription of 1 μg RNA was performed using a Transcriptor First Strand cDNA Synthesis Kit. After this, the *EGOT* relative level was tested by the lncRNA qPCR kit (SYBR Green, WH0125‐GUQ), whereas other genes at the mRNA level were measured using the ABI 7500 Fast Real‐Time PCR System (Invitrogen). A $2^{-\Delta \Delta C_{t}}$ method was used to calculate relative levels.

### Western blotting

After cells were disrupted, the total protein from cells was extracted. Protein quantification was performed using a BCA kit, and an SDS/PAGE was conducted. The protein from SDS/PAGE gel was transferred onto a poly(vinylidene difluoride) (PVDF) membrane. After this, the PVDF membrane was blocked with 5% nonfat dry milk for 2 h at room temperature. The primary antibodies we used in our research were incubated at a temperature of 4 °C overnight, whereas PVDF membranes were rinsed three times by PBST. The antibodies of Gli1 (ab134906), SMO (ab113438), PTCH1 (ab53715) and HHIP (ab39208) were provided by Abcam. After PVDF membranes were washed once more, they were incubated with the corresponding secondary antibody, whereas the membranes were washed three times with PBST. Finally, an enhanced ECL chemiluminescent kit was used to treat the membrane for color reaction.

### Statistical analysis

Data were shown as average ± standard deviation (SD). A *t*‐test, Spearman’s correlation and one‐tailed ANOVA were implemented using the latest spss, version 25.0 (IBM Corp, Armonk, NY, USA). Relative expression levels were plotted using graphpad prism 6 (San Diego, CA, USA). Quantitative data were expressed as mean ± SD based on three independent experiments. Comparisons between two groups were analyzed using Student’s *t*‐test. Multiple comparisons among groups were analyzed using Bonferroni posttests followed by two‐way ANOVA. Results were defined at a significance of *P* < 0.05.

## Results

### lncRNA *EGOT* is down‐regulated in breast cancer tissues and cell lines

**Fig. 1:**
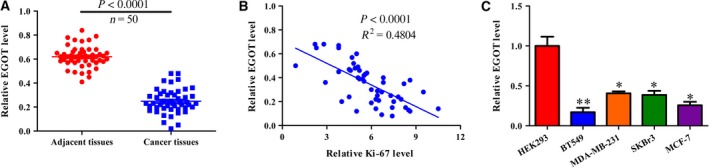
lncRNA *EGOT* is down‐regulated in breast cancer tissues and cell lines. (A) The relative *EGOT* level was measured in 50 paired breast cancer and adjacent tissues. (B) A Spearman’s correlation analysis of *EGOT* and Ki‐67 in 50 breast cancer tissues. (C) Relative *EGOT* levels were measured in breast cancer cell lines, including BT549, MDA‐MB‐231, MCF7 and SKBr3, and HEK293 was used as a control. Data shown are the mean ± SD for four independent experiments (*n* = 5) per cell line. Paired *t*‐test was performed for the comparison between two groups; linear regression analysis was performed to analyze the correlation between *EGOT* and Ki‐67. *P < 0.05; **P < 0.01 versus HEK293 cells.

The relative *EGOT* level was significantly reduced in breast cancer tissues compared with adjacent tissues (*P* < 0.0001; Fig. 1A). We examined the correlation between *EGOT* and a Ki67 expression, and discovered a negative correlation between the two genes. This most likely indicates one glaring insight: the higher the malignancy of the tumor and the larger the Ki67 value, the lower the expression of *EGOT* (*P* < 0.0001; Fig. 1B). The quantitative real‐time PCR (qRT‐PCR) results exhibited that the relative level of *EGOT* expression was significantly down‐regulated in breast cancer cell lines BT549, MDA‐MB‐231, MCF7 and SKBr3, compared with control cell lines in HEK293 (Fig. 1C).

### Overexpression of *EGOT* impedes cell proliferation and migration

**Fig. 2:**
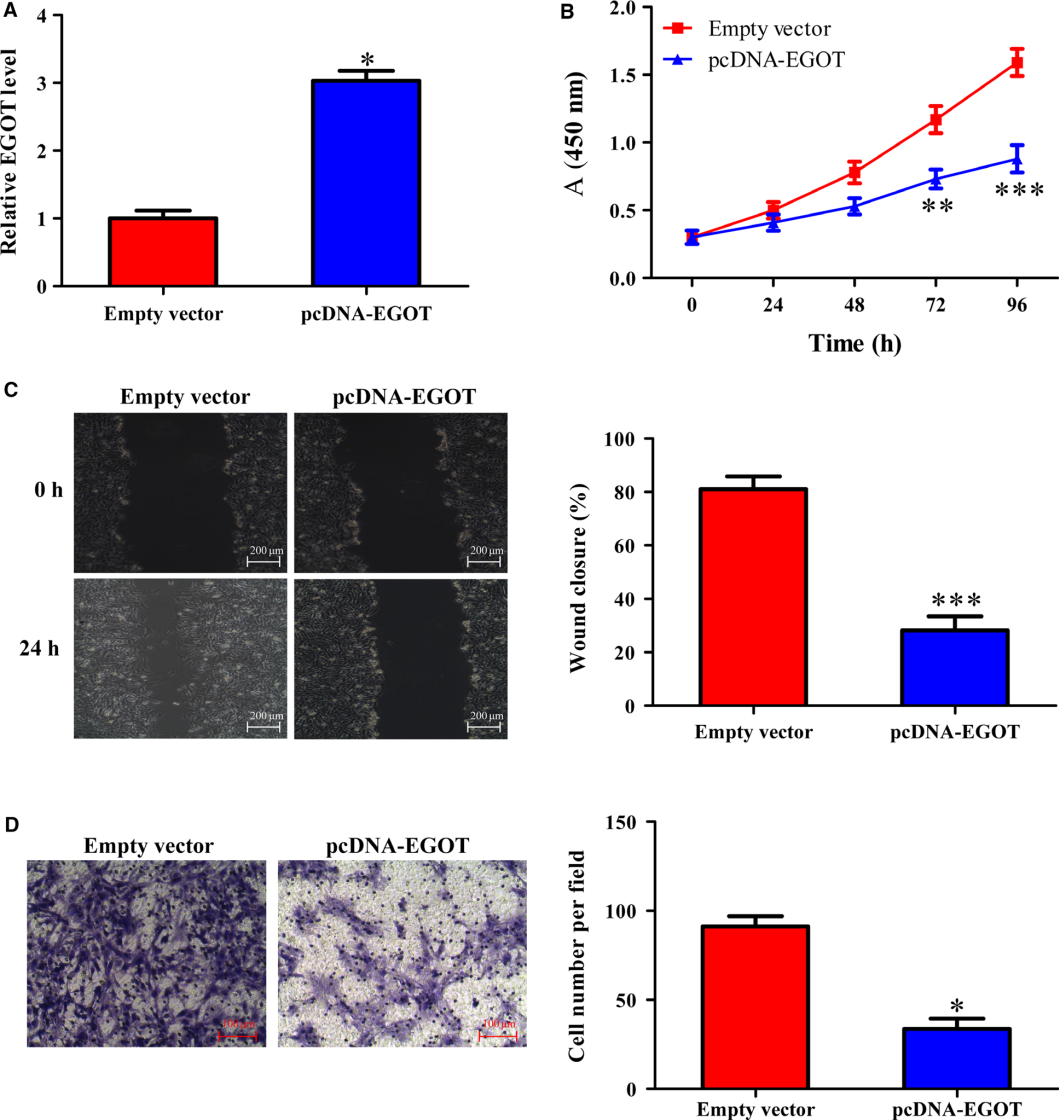
Overexpression of *EGOT* affects cell viability and migration. (A) The relative level of *EGOT* in cells transfected with pcDNA–EGOT or pcDNA was detected by qRT‐PCR. (B) An MTT assay was conducted to assess cell viability. (C, D) Wound healing assays and Transwell migration assays were used to detect cell migration after an overexpression of *EGOT*. Scale bars: 200 μm (C); 100 μm (D). The results were the average from at least three independent experiments, mean ± SD; paired *t*‐test was performed for the comparison between two groups; Bonferroni posttests followed by two‐way ANOVA were used to assess the cell viability. *P < 0.05; **P < 0.01; ***P < 0.001 versus pcDNA.

The expression of *EGOT* from BT549 was the lowest among these breast cancer cell lines, with BT549 being used for further functional experiments. When the *EGOT* overexpression system was established, results from the pcDNA–EGOT transfection revealed that *EGOT* was clearly enhanced in the pcDNA–EGOT group compared with the control group (Fig. 2A). MTT assays were conducted to evaluate cell viability, with results revealing that cell viability is greatly inhibited in the pcDNA–EGOT group compared with pcDNA (Fig. 2B). Cell migration was then detected using a Transwell migration and wound healing assay, with results revealing that the migration ability of cells transfected with pcDNA–EGOT was tremendously impaired compared with the pcDNA group (Fig. 2C,D).

### Gli1 is up‐regulated in the breast cancer tissues, and Gli1, SMO, PTCH1 and HHIP were all down‐regulated after an EGOT overexpression

**Fig. 3:**
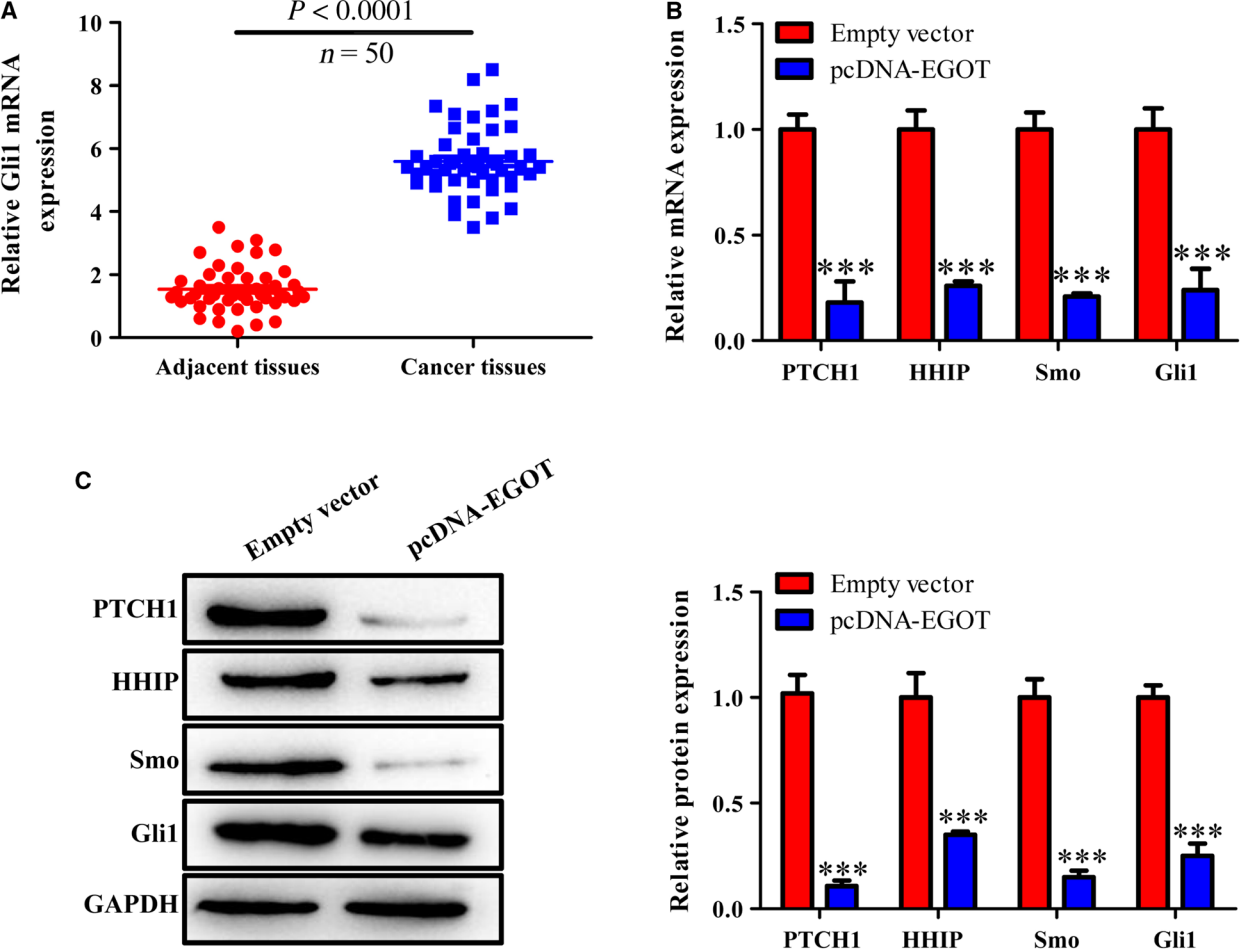
Gli1 is up‐regulated in breast cancer tissues, and Gli1, SMO, PTCH1 and HHIP were down‐regulated after *EGOT* overexpression. (A) The relative Gli1 mRNA level was measured by qRT‐PCR in 50 breast cancer and adjacent tissues. (B) The expression of Gli1, SMO, PTCH1 and HHIP at mRNA levels was detected in cells transfected with pcDNA–EGOT or pcDNA. (C) The expression of Gli1, SMO, PTCH1 and HHIP at protein level was detected in cells transfected with pcDNA–EGOT or pcDNA. The results were the average from at least three independent experiments, mean ± SD; paired *t*‐test was performed for the comparison between two groups. ***P < 0.001 versus pcDNA.

Interestingly, we found that Gli1 was significantly elevated at the mRNA level in breast cancer tissues compared with adjacent tissues (Fig. 3A). Not only this, but the expression of Gli1, SMO, PTCH1 and HHIP at mRNA and protein levels was detected in either the pcDNA–EGOT or pcDNA group (Fig. 3B,C). These results demonstrated that Gli1, SMO, PTCH1 and HHIP were all reduced at transcriptional and translational levels in cells transfected with pcDNA–EGOT. We therefore speculate that lower *EGOT* expression levels may be related to the Hh pathway.

### Purmorphamine restores the expression of key genes in the Hh signaling pathway in breast cancer cell line BT549 overexpressing *EGOT*

**Fig. 4:**
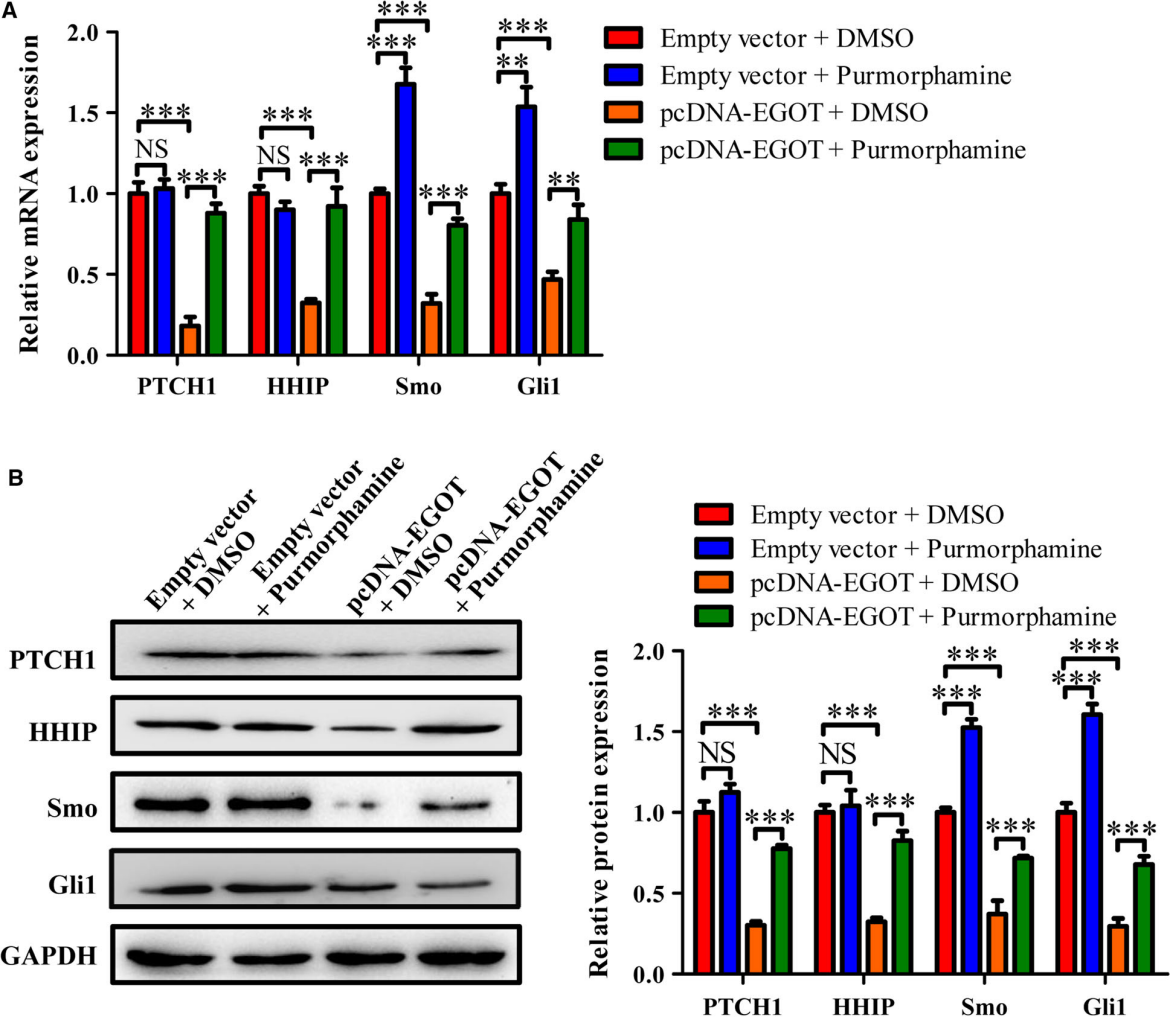
Effects of *EGOT* overexpression on the Hh signaling pathway‐related genes in breast cancer cell line BT549. BT549 cells were transfected with pcDNA–EGOT or pcDNA, and then treated with purmorphamine or DMSO. (A) The relative *EGOT* level was measured by qRT‐PCR in various treatment groups. (B) The expression of Gli1, SMO, PTCH1 and HHIP at protein levels was detected in these groups. The results were the average from at least three independent experiments, mean ± SD; Bonferroni posttests followed by two‐way ANOVA were used to compare the four groups. **P < 0.01; ***P < 0.001. NS, not significant.

We further discovered that validating the role of the Hh pathway in *EGOT* overexpressions triggered inhibitive effects on cell viability and migration. Purmorphamine, the agonist of the Hh pathway, was used to treat cells with an overexpression of *EGOT*. The results showed that there was no significant difference at mRNA and protein levels in PTCH1 and HHIP in the pcDNA group with or without purmorphamine molecules. The mRNA and protein levels of Gli1 and SMO were up‐regulated with purmorphamine in the pcDNA empty vector group. However, there was an obvious increase in mRNA and protein levels in PTCH1, Gli1, SMO and HHIP in pcDNA–*EGOT* cells with purmorphamine compared with those who had only pcDNA–EGOT cells (Fig. 4A,B).

### Purmorphamine restores viability and migration in breast cancer cell line BT549, with an overexpression of *EGOT*

**Fig. 5:**
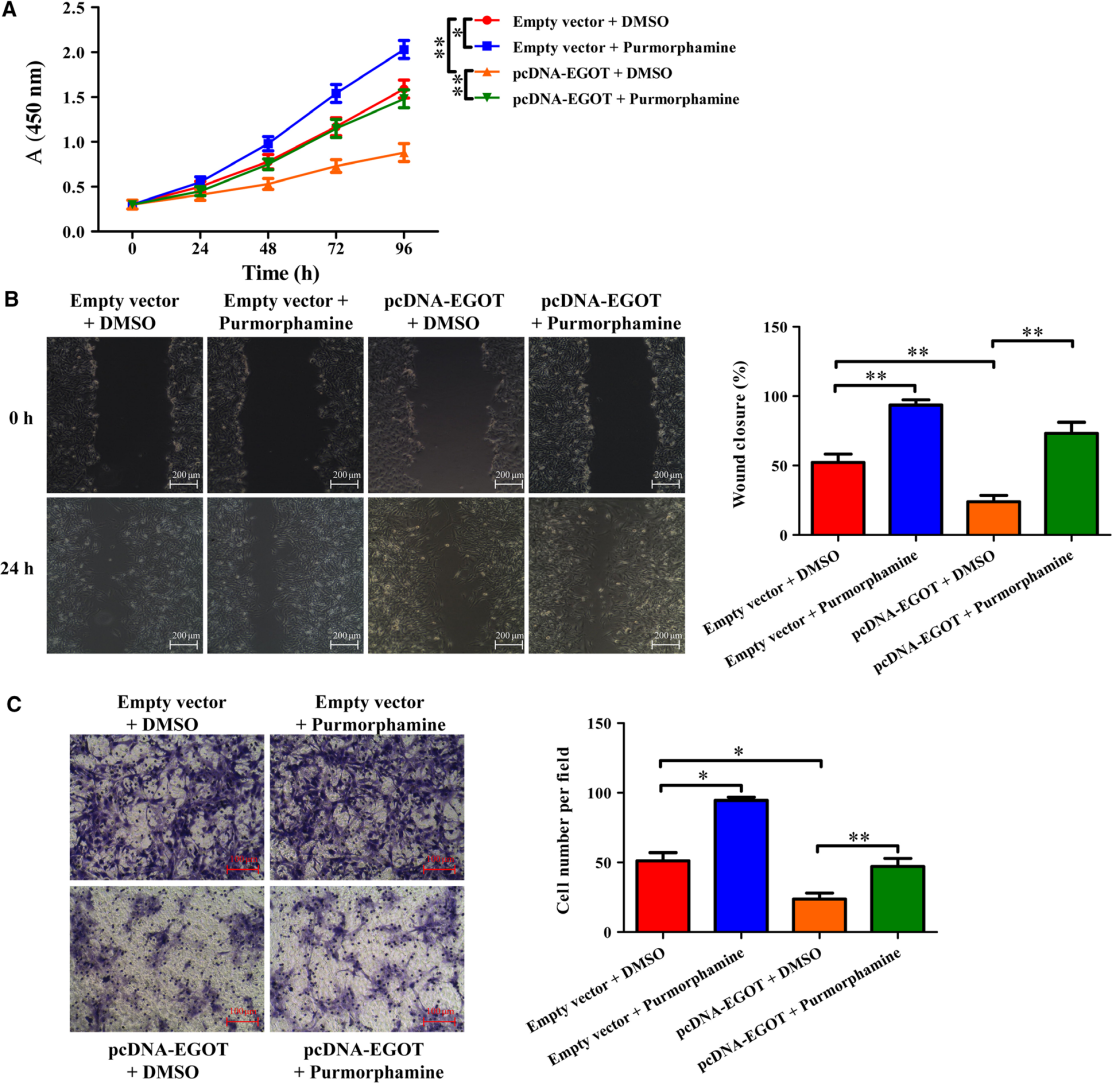
Effect of Hh signaling pathway on cell viability and migration in breast cancer cell line BT549. BT549 cells were transfected with pcDNA–EGOT or pcDNA, then treated with purmorphamine or DMSO. (A) An MTT assay was used to measure cell viability in various treatment groups. (B, C) Wound healing (B) and Transwell assays (C) were used to detect cell migration in these groups. Scale bars: 200 μm (B); 100 μm (C). The results were the average from at least three independent experiments, mean ± SD; paired *t*‐test was performed for the comparison between two groups; Bonferroni posttests followed by two‐way ANOVA were used to assess cell viability. *P < 0.05; **P < 0.01.

As shown in Fig. 5A, the viability of cells transfected with pcDNA–EGOT and incubated with DMSO pcDNA was lower than that in pcDNA + DMSO groups, although the viability of these cells enhanced after cells with *EGOT* overexpression were treated with purmorphamine. Moreover, the results of cell viability and migration showed a similar tendency to the cell viability during two other methods of treatment: the wound healing test (Fig. 5B) and the Transwell assay (Fig. 5C).

## Discussion

With advances in RNA sequencing and transcriptome analysis, researchers have surprisingly found that up to 76% of the human genome can be transcribed into RNA molecules, including lncRNAs [24, 25]. According to the latest version of lncRNA database LNCipedia 5.2, there are 127 802 transcripts and 56 946 human annotated lncRNAs [26]. In the past two decades, lncRNAs have been discovered to be aberrantly expressed, regulating the development and progression of tumors [27, 28]. A number of lncRNAs have been deregulated in cancers, controlled by epigenetic, genetic and transcriptional factors [29]. Up until to now, moreover, numerous abnormal expression lncRNAs have been found in breast cancer that are specifically involved in the cancer’s initiation and development [30, 31, 32, 33, 34].

In our study, lncRNA *EGOT* was detected in breast cancer tissues and cell lines (Fig. 1), which was further consistent with Xu *et al.*’s results [19], whereby the expression of *EGOT* was negatively correlated with Ki67. As expected, overexpression in *EGOT* negatively affected cell proliferation and migration (Fig. 2). Therefore, the role of *EGOT* in breast cancer progression has been identified as a tumor suppressor.

In recent publications, aberrant expressed lncRNAs were reported to strengthen cancer progression via activation of the Hh signaling pathway, including gastric cancer, prostate cancer, breast cancer and pancreatic cancer [16, 18, 35, 36]. To further uncover the role of *EGOT* in breast cancer, we studied the relationship between *EGOT* and the Hh signaling pathway. As the terminal effector of the typical Hh signaling pathway, the Gli1 family functions as a transcription factor, and abnormal regulation of Gli1 protein leads to tumorigenesis [17, 37]. Interestingly, we found that Gli1 was significantly up‐regulated in breast cancer tissues, whereas PTCH1, SMO, Gli1 and HHIP at mRNA and protein levels were all down‐regulated after controlling for *EGOT* overexpression (Fig. 3). We hypothesize that an overexpression of *EGOT* decreased the expression of PTCH1, thus suppressing its binding to the Hh ligand, which has proved to lead to low expressions of SMO and Gli1. As a result, the expression of the Hh interacting protein HHIP in nucleus was down‐regulated. According to these results, we deduce that the Hh signaling pathway may be involved in breast cancer progression.

Onishi and Katano [38] and Rubin and de Sauvage [14] have concluded that abnormal activation in the Hh signaling pathway is closely correlated with the initiation and development of various cancers. Purmorphamine is an Hh agonist that directly targets SMO transmembrane proteins [39]. Activation of Hh signaling by purmorphamine promotes the transcription of various genes, including Gli1, PTCH1 and alkaline phosphatase [34]. To confirm whether purmorphamine can reverse the expression of genes in the Hh signaling pathway caused by an overexpression in *EGOT*  in breast cancer, we transfected BT549 cells with pcDNA–EGOT or pcDNA. After this, cells were treated with purmorphamine. The results demonstrate that purmorphamine reversed the expression of PTCH1, SMO, Gli1 and HHIP in the overexpression system of EGOT (Fig. 4), which was consistent with the finding by Lin *et al.* [34]. Purmorphamine inhibited osteoblast differentiation in human multipotent adipose‐derived stem cells and mesenchymal stem cells (MSCs) from bone marrow by activating the Hh signaling pathway [40]. Purmorphamine was then used to measure cell proliferation and migration ability in the overexpression system of *EGOT*. Results indicated that purmorphamine reversed cell proliferation and migration abilities in breast cancer (Fig. 5).

According to the expression of estrogen receptor (ER), progesterone receptor and human epidermal growth factor receptor 2 (HER‐2), breast cancer is identified as four molecular subtypes: basal‐like, HER‐2 positive, luminal A and luminal B, with the first two subtypes from negative ER tumors and the last two from positive ER tumors [41]. The basal‐like tumors are mainly composed of triple‐negative breast cancer (TNBC), tumors that lack expression of ER, progesterone receptor and HER‐2 [42]. We collected a total of 50 breast cancer samples, 18 of which were TNBC tumors, 9 HER‐2 tumors, 13 luminal A tumors and 10 luminal B tumors. TNBC takes up 15–20% in all invasive breast cancers and occurs more frequently in young women; meanwhile, the survival rate of TNBC is the worst [43]. In our study, BT549 and MDA‐MB‐231 cells belong to the TNBC subtype, whereas MCF7 is a luminal A subtype and SKBr3 is an HER‐2‐positive subtype. In experiments involved in the Hh signaling pathway, the cell line BT549 was used to further real molecular mechanisms in this aggressive subtype of breast cancer.

## Conclusions

*EGOT* was greatly down‐regulated in breast cancer tissues and cell lines, and the relative level of *EGOT* was negatively correlated with the expression of Ki67. As we inferred, the overexpression of *EGOT* impaired cell viability and migration in BT549 cell lines. Furthermore, the expression of Gli1 was significantly increased in breast cancer tissues. The relative expressions of PTCH1, SMO, Gli1 and HHIP at both mRNA and protein levels were reduced in the overexpression of *EGOT*. Purmorphamine molecules were used to treat cells with an overexpression of *EGOT* to ensure two things: first, that the Hh pathway was activated; and second, that the roles of Hh signaling in *EGOT*‐induced inhibitive effects on breast cancer cells were found. The results found that the relative expression of PTCH1, SMO, Gli1 and HHIP, and cell viability and migration caused by an overexpression of *EGOT* were reversed by purmorphamine. In total, lncRNA *EGOT* is proved to inhibit TNBC cell viability and migration via modulation of the Hh pathway. Our present research supports helpful evidence to the anti‐oncogene of *EGOT* in breast cancer and may extend novel knowledge of therapeutic methods for TNBC.

## Conflict of interest

The authors declare no conflict of interest.

## Author contributions

SQ and GBC conducted experiments and were responsible for data acquisition and manuscript writing. JP, JL and JMC were responsible for data interpretation and data analysis. JJW and LL helped in statistical analysis. KXY conceived and designed the study, and revised the manuscript critically for important intellectual content. All authors read and approved the final manuscript.
